# Anti-aging effects of *Ribes meyeri* anthocyanins on neural stem cells and aging mice

**DOI:** 10.18632/aging.103955

**Published:** 2020-09-12

**Authors:** Jiaming Gao, Yating Wu, Dajun He, Xiaoqi Zhu, Hongbin Li, Haifeng Liu, Hailiang Liu

**Affiliations:** 1 Institute for Regenerative Medicine, Shanghai East Hospital, Tongji University School of Medicine, Shanghai 200123, China; 2 Key Laboratory of Xinjiang Phytomedicine Resource and Utilization of Ministry of Education, College of Life Sciences, Shihezi University, Shihezi 832003, China; 3 China Colored-Cotton (Group) Co., Ltd., Urumqi 830014, Xinjiang, China

**Keywords:** aging, *Ribes meyeri anthocyanin*, naringenin, cognition, senescence

## Abstract

Aging is associated with neurological impairment and cognitive decline. Flavonoids are very promising in anti-aging research in mouse models. *Ribes meyeri* anthocyanins are rich in abundant flavonoids, but their anti-aging biological activities remain unknown. In this study, we prepared an *R. meyeri* anthocyanin extract and analyzed its effects on neural stem cell (NSC) senescence *in vivo* and *in vitro*. We isolated mouse NSCs and used cell counting kit-8 (CCK-8), cell cycle, reactive oxygen species (ROS), and immunofluorescence methods to analyze the anti-aging effects of *R. meyeri* anthocyanins as well as naringenin (Nar), which metabolic analysis revealed as an important flavonoid in *R. meyeri* anthocyanins. RNA-sequencing (RNA-seq) and enzyme-linked immuno sorbent assay (ELISA) methods were also used to investigate Nar-specific mechanisms of anti-aging. After *R. meyeri* anthocyanin treatment, NSC proliferation accelerated, and NSCs had decreased senescence markers, and reduced P16^ink4a^ expression. *R. meyeri* anthocyanin treatment also reversed age-dependent neuronal loss *in vivo* and *in vitro*. Nar blocked mNSC aging *in vitro* and improved spatial memory and cognitive abilities in aging mice through downregulation of plasma TNF-α protein. These findings suggest that *R. meyeri* anthocyanins increase NSC proliferation and improve neurogenesis with aging via Nar-induced reductions in TNF-α protein levels *in vivo*.

## INTRODUCTION

*Ribes meyeri* (also known as *R. meyeri* Maxim) belongs to the Saxifragaceae family [1, 2]. The roots and leaves of *R. meyeri* are rich in a class of plant secondary metabolite flavonoids [1–6] with multiple functions, including anti-inflammatory and anti-cancer effects [7–9]. Zhou et al. first isolated naringenin (Nar) from *R. meyeri* anthocyanins [1]. Nar is a main component of *R. sativum*; however, the anti-aging biological activity of Nar and *R. meyeri* anthocyanins remains unknown.

Stem cell exhaustion is an important hallmark of aging [10]. An age-dependent loss of neural stem cells (NSCs) is associated with cognitive decline, and NSCs are involved in diseases associated with memory loss [11, 12]. There is a marked drop in NSC and neuronal numbers in the aging murine brain [13]. Causes of aging in stem cells include shortened telomeres, increased levels of reactive oxygen species (ROS), activation of the senescence marker P16^ink4a^, and cell cycle arrest [14–16]. These factors ultimately lead to nervous system damage, metabolic disorders, and cognitive dysfunction [17].

Considering that these changes are related to aging, the functions of *R. meyeri* anthocyanins have not been well studied. Accordingly, we investigated the effects of *R. meyeri* anthocyanins on NSCs in aging mice. Our results show that *R. meyeri* anthocyanins improve the aged phenotype by reversing senescence of mNSCs *in vitro*, as well as by improving cognitive levels and enhancing NSC proliferation and neuronal numbers in aging mice. We also revealed that the flavonoid Nar, which is enriched among *R. meyeri* anthocyanins, reverses aging in NSCs and improves cognitive levels in aging mice by inhibiting the tumor necrosis factor (TNF) signaling pathway.

## RESULTS

### Effects of *R. meyeri* anthocyanins on age-related changes in mNSCs

To determine the effects of *R. meyeri* anthocyanins on cell senescence, we used NSCs from 23-month-old mice (23M-mNSCs). Considering that *R. meyeri* anthocyanins may exert cytotoxic or protective effects on cells depending on their concentration, we first needed to ascertain a safe concentration of *R. meyeri* anthocyanins to treat 23M-mNSCs. The viability of cells treated with *R. meyeri* anthocyanins was detected using a cell counting kit-8 (CCK-8) assay. At concentrations up to 100 pg/mL, *R. meyeri* anthocyanin treatment for 48 or 72 h increased 23M-mNSC cell viability (Supplementary Figure 1A). Accumulation of ROS during aging plays a major role in mitochondrial damage [16, 18]. To determine whether *R. meyeri* anthocyanins can suppress oxidative damage, we performed ROS assays. *R. meyeri* anthocyanins inhibited oxidative damage at a concentration of 100 pg/mL (Supplementary Figure 1B). Compared with controls, we observed marked changes in proliferation and ROS levels in *R. meyeri* anthocyanin-treated cells, demonstrating that 100 pg/mL *R. meyeri* anthocyanins stimulate 23M-mNSC proliferation and inhibit oxidative damage.

**Figure 1:**
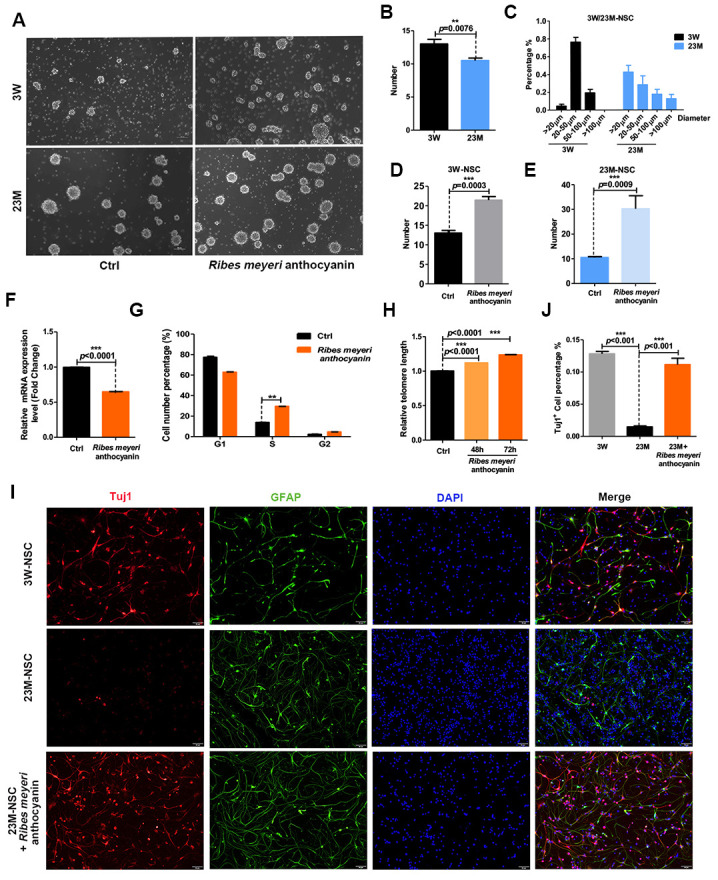
***Ribes meyeri* anthocyanins rescue the senescence phenotype of mouse neural stem cells (mNSCs).** (**A**) Neurospheres clonally derived from 23-month-old (23M-NSC) and 3-week-old (3W-NSC) mouse brains and treated with 100 pg/mL *R. meyeri* anthocyanins. (**B**) Differences in neurosphere numbers between 23M-NSC and 3W-NSC. (**C**) The shape of 3W-NSC neurospheres was more uniform, with an increased number and size (20–50 μm) compared with 23M-NSC neurospheres. (**D**, **E**) Treatment with *R. meyeri* anthocyanins increased the numbers of both 3W-NSC- and 23M-NSC-derived neurospheres compared with controls. (**F**–**H**) In 23M-NSCs treated with 100 pg/mL *R. meyeri* anthocyanins for 48 h, p16^ink4a^ mRNA expression, cell cycle, and relative telomere lengths were measured. (**F**) Cell senescence marker p16^ink4a^ mRNA expression detected by qRT-PCR. (**G**) Cell cycles were determined using flow cytometry. (**H**) qRT-PCR detection of relative telomere length. (**I**) Immunofluorescence staining of TuJ1 and GFAP in 23M-NSCs treated with *R. meyeri* anthocyanins and control. (**J**) Quantification of (**I**). Data are presented as the mean ± SD of three independent experiments. *P < 0.05, **P < 0.01, and ***P < 0.0001 compared with untreated cells.

To determine the effects of *R. meyeri* anthocyanins on neurosphere formation, we isolated NSCs from the brains of 3-week-old (3W-mNSCs) and 23-month-old (23M-mNSCs) mice. The NSCs were subcultured and treated with 100 pg/mL *R. meyeri* anthocyanins (Figure 1A). Compared with the 23M-mNSCs, the number of neurospheres in the 3W-mNSC group was increased, and the shapes of the neurospheres were more uniform and rounded, with an increased diameter of 20–50 μm (Figure 1B–1D). In addition, the neurospheres derived from 23M-mNSCs after 5 days were smaller in size (20 μm), and this finding was consistent with results from previous studies [19, 20]. When treated with *R. meyeri* anthocyanins, neurosphere formation was significantly increased in both 3W-mNSCs and 23M-mNSCs (Figure 1E). Senescence is closely related to cell cycle arrest, shortened telomeres, and increased expression of the senescence gene p16^ink4a^. *R. meyeri* anthocyanin-treated 23M-NSCs had significantly reduced p16^ink4a^ gene expression, a greater number of cells in the S phase, and lengthened telomeres compared with untreated cells (Figure 1F–1H). Moreover, we know that aging reduces the proliferation of NSCs and reduces their differentiation capabilities for neuronal production [13, 21]. Upon differentiation of 23M-mNSCs, there was a significant increase in the number of TuJ1-positive neurons after *R. meyeri* anthocyanin treatment (Figure 1I–1J), indicating that *R. meyeri* anthocyanin treatment promotes the differentiation of NSCs into neurons. These results demonstrate that *R. meyeri* anthocyanins have a positive impact on aged NSCs *in vitro*.

### *R. meyeri* anthocyanin treatment improves learning and memory abilities of aging mice

**Figure 2:**
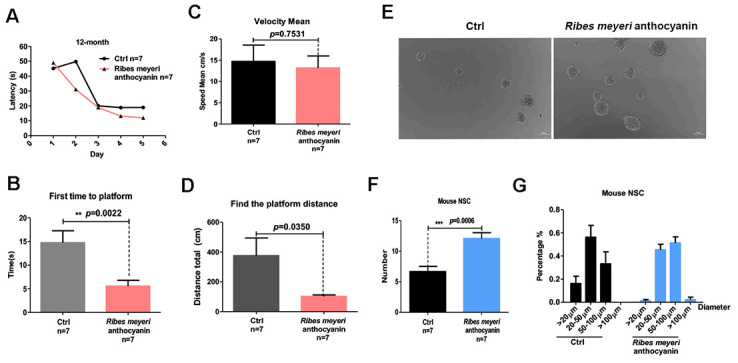
***Ribes meyeri* anthocyanins enhance mouse memory and learning abilities by increasing neural stem cells (NSCs).** (**A**) Time taken to find the platform. (**B**) Time taken to first reach the platform. (**C**) Mean velocity to find the platform. (**D**) Distance swum before finding the platform. (**E**, **F**) Treatment with *R. meyeri* anthocyanins increased neurosphere number and size compared with controls. (**G**) Neurospheres of 50–100 μm in size were increased in number by *R. meyeri* anthocyanin treatment in mice. Data are presented as the mean ± SD of three independent experiments. *P < 0.05, **P < 0.01, and ***P < 0.0001 compared with untreated cells. “*n*” indicates the number of animals in each experimental group.

Aging is associated with cognitive decline [22, 23]. We therefore evaluated the effects of *R. meyeri* anthocyanins on the learning and memory abilities of mice. The Morris water maze (MWM) test revealed that, when adult C57BL/6 mice (12 months old) were administered 100 mg/kg *R. meyeri* anthocyanins for 2 months, the average time to reach the platform was decreased compared with controls (Figure 2A). We also examined the time taken to first reach the platform (Figure 2B). The *R. meyeri* anthocyanin-treated group took significantly less time than the control group, although the average speed was not significantly different between the two groups (Figure 2C), suggesting that *R. meyeri* anthocyanins have little effect on the motor ability of mice. Furthermore, in the treated group, the mice swam a significantly shorter distance before reaching the platform area compared with the untreated group (Figure 2D). These results demonstrate that *R. meyeri* anthocyanins can improve the cognitive and spatial memory abilities of aging mice.

Aging-associated cognitive decline has been linked to reduced NSC numbers [11, 24]. Accordingly, we isolated and cultured NSCs from the mouse brain subventricular zone (SVZ) after intragastric administration of 100 mg/kg *R. meyeri* anthocyanins (Figure 2E). The resulting numbers of neurospheres were measured, and there were significantly more neurospheres in the *R. meyeri* anthocyanin-treated group compared with the control group (Figure 2F). Next, we analyzed neurosphere size distribution. *R. meyeri* anthocyanin-treated mouse neurospheres were primarily in the upper size range (50–100 μm diameter), whereas control neurospheres were more commonly in the lower range (20–50 μm diameter) (Figure 2G).

To further verify the influence of *R. meyeri* anthocyanin treatment on NSCs and neurons *in vivo*, immunofluorescence staining of the hippocampus was performed and the percentages of immunopositive cells were analyzed. Using the well-known NSC markers nestin and Ki67 [25, 26], we revealed an increase in nestin- and Ki67-positive cells in the hippocampal region (Supplementary Figure 2A, 2B) of *R. meyeri* anthocyanin-treated mice. In addition, the number of TuJ1-positive cells (a marker of mature neurons) was also increased in these mice (Supplementary Figure 2C, 2D). Together, these results indicate that 100 mg/kg *R. meyeri* anthocyanins can improve spatial memory and cognitive abilities in aging mice by promoting NSC proliferation and increasing neuronal numbers.

### Nar rescues senescent phenotypes of 23M-NSCs

**Figure 3:**
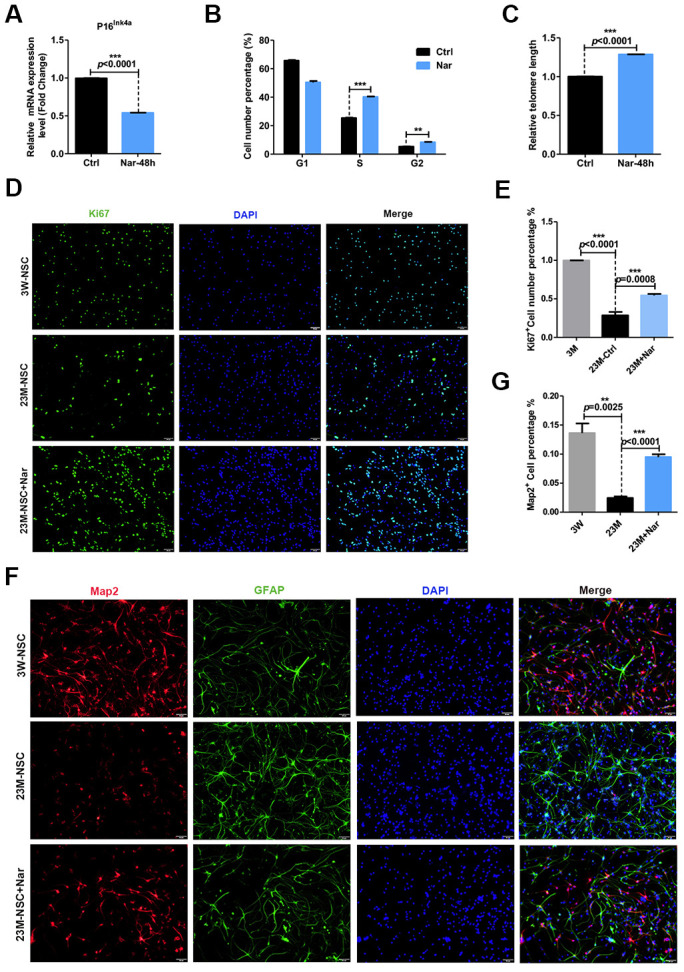
**Effect of naringenin (Nar) on senescence of mouse neural stem cells (mNSCs).** (**A**) p16^ink4a^ mRNA expression was measured by qRT-PCR in 23M-NSCs with and without treatment of 6.8 μg/mL Nar for 48 h. (**B**) Cell cycle phase distributions of 23M-NSCs treated with Nar for 48 h and control cells. (**C**) The relative telomere length of 23M-NSCs increased significantly with Nar treatment. (**D**) Immunofluorescence Ki67 staining of mNSCs treated with Nar for 48 h, with DAPI nuclear labeling. (**E**) Quantification of (**D**). (**F**) Representative fields of MAP2 and GFAP immunofluorescence staining of cultured mNSCs after control and Nar treatment. (**G**) Quantification of (**F**). Data are presented as the mean ± SD of three independent experiments. *P < 0.05, **P < 0.01, and ***P < 0.0001 compared with untreated cells.

*R. meyeri* anthocyanins are rich in many kinds of flavonoids and bioactive components [1, 3–5]. To determine which component of *R. meyeri* anthocyanins plays an important role in anti-aging, we performed a metabolomic analysis of *R. meyeri* anthocyanins. We revealed that Nar may be an important flavonoid metabolite because the flavonoid biosynthetic pathway was highly enriched in *R. meyeri* anthocyanins (Supplementary Table 1). To determine the anti-senescent effects of Nar, various concentrations (0, 1.7, 3.4, 6.8, 13.6, and 27.2 μg/mL) were applied to 23M-NSCs and CCK-8 assays were used to investigate cell viability. As Supplementary Figure 3 shows, 6.8 μg/mL Nar treatment increased cell viability by 150%. To further explore the anti-senescent effects of Nar, 23M-NSCs were treated with Nar, and aging-related indicators were then detected. In 23M-NSCs treated with 6.8 μg/mL Nar, p16^ink4a^ mRNA expression was reduced (Figure 3A). Nar also lengthened the telomeres of cultured mNSCs and increased DNA synthesis (Figure 3B, 3C). To further explore the effects of Nar on cell proliferation, we performed immunofluorescence staining of Ki67, which is a nuclear antigen of mitotic cells that is expressed throughout the cell cycle, except in G0 [19, 27]. Treatment with Nar enhanced cell proliferation, as measured by increased numbers of Ki67-positive cells (Figure 3D, 3E). Next, we also detected the differentiation potential of NSCs into neurons. Indeed, 6.8 μg/mL Nar treatment also increased the number of MAP2-positive cells (Figure 3F, 3G). These results consistently demonstrate that Nar can reverse 23M-NSC senescence.

### Nar improves memory and cognition in aging mice

**Figure 4:**
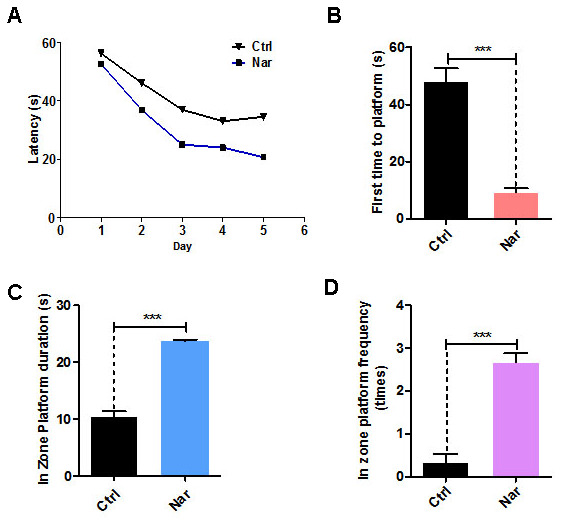
**Naringenin (Nar) enhances memory and learning abilities in aging mice.** (**A**) Escape latency of Nar-treated aging mice was shorter compared with the control group. (**B**) Time taken to first reach the platform was shorter in Nar-treated aging mice than in the control group. (**C**) Duration of time spent in the platform zone. (**D**) Frequency of being observed in the platform zone for Nar-treated aging mice compared with the control group. Data are presented as the mean ± SD of three independent experiments. *P < 0.05, **P < 0.01, and, ***P < 0.0001 compared with the control group. “*n*” indicates the number of animals used for each experimental group.

Cognitive levels decline with aging, and anti-aging treatments can improve cognitive levels [11, 28]. To verify the anti-aging effects of Nar *in vivo*, we injected 20 mg/kg Nar into aging C57BL/6 mice (12 months old) through the tail vein. The MWM test was used to examine the spatial memory and cognitive abilities of the mice. Over 5 days of repeated training, the time taken for Nar-treated aging mice to find the platform gradually decreased compared with the control group (Figure 4A). The test results revealed that the time taken to first reach the platform was reduced (Figure 4B), while the time spent in the platform zone was increased (Figure 4C). Furthermore, Nar-treated aging mice were observed within the platform zone at a higher frequency than the control group (Figure 4D). Together, these results suggest that Nar can improve spatial memory and cognitive abilities in aging mice.

### RNA sequencing analysis of Nar-treated blood from aging mice

**Figure 5:**
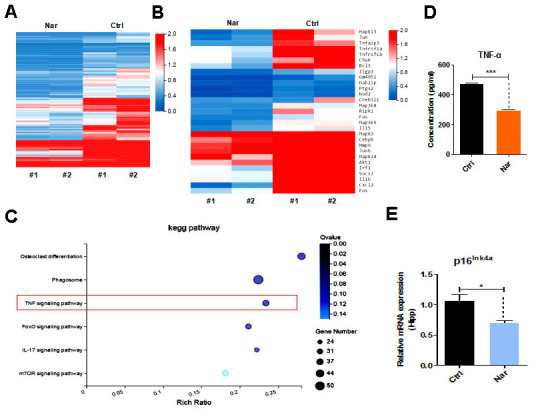
**Tumor necrosis factor (TNF)-α plays an essential role in naringenin (Nar)-mediated anti-aging effects.** (**A**) Summary of gene expressions in mouse blood, as determined by RNA-seq. (**B**) Heatmap showing differentially expressed genes (DEGs) in the blood between control and Nar-treated mice. DEGs were identified by a fold change of > 2.0 or < 0.5. (**C**) Bar graph showing the major correlated signaling pathways. (**D**) Heatmap showing DEGs in the TNF signaling pathway (**E**) Plasma TNF-α levels determined by enzyme-linked immunosorbent assay. (**E**) p16^ink4a^ gene expression levels, as determined by qRT-PCR. Data are presented as the mean ± SD of three independent experiments. *P < 0.05, **P < 0.01, and ***P < 0.0001 compared with the control group.

To investigate the Nar-specific anti-aging mechanisms, we analyzed the gene expression profile of Nar-treated blood from aging mice using RNA sequencing (RNA-seq). The results revealed 1,961 differentially expressed genes (DEGs). Generally, Nar downregulated gene expression, and only a small number of genes were upregulated compared with the untreated control group (Figure 5A, 5B). Additionally, important signaling pathway genes related to aging were downregulated, especially those in the TNF signaling pathway (Figure 5C). These results imply that Nar affects the TNF signaling pathway, thus profoundly influencing the function of TNF-α, which is involved in aging-related cognitive impairment [29, 30]. We therefore examined TNF-α levels in the plasma of mice. Results from enzyme-linked immunosorbent assays (ELISA) revealed that Nar treatment led to the downregulation of plasma TNF-α levels (Figure 5D). We also examined p16^ink4a^ gene expression in the hippocampus of mice, and demonstrated that Nar treatment downregulates p16^ink4a^ mRNA expression (Figure 5E).

## DISCUSSION

Aging is associated with an increased risk of cognitive decline as well as a loss of learning and memory ability [22, 23]. Neuronal loss and impaired neurogenesis are closely related to age-associated brain dysfunction [11–13]. Thus, increasing the number of neurons and improving neurogenesis is crucial for anti-aging treatment. *R. meyeri*, a plant rich in flavonoids and other bioactive substances [1, 3–6], has many functions that have not yet been described.

In the present study, *R. meyeri* anthocyanins promoted mNSC proliferation and increased the formation of neurospheres with uniform morphology. *R. meyeri* anthocyanin treatment also improved the cellular aging phenotype, with reduced levels of ROS and aging-related P16^ink4a^ gene expression, increased DNA synthesis phase, and lengthened telomeres. Moreover, immunofluorescence staining results revealed that *R. meyeri* anthocyanins stimulated the formation of neurons. These results suggest that *R. meyeri* anthocyanins have anti-aging effects on mNSCs, and may also directly promote the formation of neurons *in vitro*. Furthermore, cognitive decline is closely related to aging [22, 23]. Our MWM results demonstrated that intragastric administration of *R. meyeri* anthocyanins improved spatial memory and learning abilities in aging mice, and also increased mNSC and neuronal numbers in the hippocampus of aging mice. These results suggest that *R. meyeri* anthocyanins may enhance cognitive function by increasing mNSC proliferation and neurogenesis.

The use of metabolomic analysis to investigate the specific composition of *R. meyeri* anthocyanins revealed that Nar may be an important flavonoid metabolite. Nar has previously been reported to ameliorate myocardial cell senescence [31], improve the metabolic capacity of the intestinal tract [32], and exert anti-inflammatory [33] and anti-cancer [34] effects. However, the effects of Nar on NSCs during aging remains unknown. To explore the anti-aging effects of *R. meyeri* anthocyanins, we conducted further studies using Nar. Treatment with 6.8 μg/mL Nar increased cell viability, reduced P16^ink4a^ gene expression, lengthened telomeres, and promoted mNSC differentiation into neurons *in vitro*. To further assess the effects of Nar on cell proliferation, we performed immunofluorescence studies. Results indicated that Nar treatment increased both the number of Ki67-positive cells and the proportion of MAP2-positive cells, suggesting that Nar may promote neurogenesis. Furthermore, the effects of Nar on learning and memory were also evaluated in aging mice. The MWM test results consistently demonstrated that Nar treatment enhances spatial learning in aging mice. Interestingly, RNA-seq analysis revealed that Nar may affect senescence via the TNF signaling pathway, especially by downregulating TNF-α expression in the blood of aging mice. ELISA assays also indicated that Nar treatment reduced plasma TNF-α levels compared with control aging mice. TNF-α is a key factor in the TNF signaling pathway and is closely related to cognitive aging. Its functions include the promotion of pathological changes in hippocampal synapses [29] and the inhibition of precursor cell proliferation [35]. Altered TNF levels are associated with cognitive impairment in depression, schizophrenia, bipolar disorder, and Alzheimer's disease [30, 36, 37]. More specifically, TNF-α is upregulated in patients with Alzheimer’s disease [38].

In summary, our study demonstrates that *R. meyeri* anthocyanins improve the effects of aging in NSCs via Nar, which downregulates TNF-α levels *in vivo* and improves cognition in aging mice. Collectively, our findings provide a novel strategy for the development of clinical treatments, aimed at greater realization of the medicinal value of *R. meyeri* anthocyanins.

## MATERIALS AND METHODS

### Experimental animals

This study was approved by the Tongji University Animal Care Committee and conducted in accordance with institutional guidelines. *R. meyeri* anthocyanins were administrated intragastrically for 2 months to 12-month-old mice maintained on the C57BL/6 background. Nar was injected into caudal vein for 1 month to 12- month-old mice maintained on the C57BL/6 background. Control mice were given the same volume of phosphate-buffered saline (PBS).

### Preparation of *R. meyeri* anthocyanins and Nar

*R. meyeri* fruit (500 g) was homogenized using a tissue homogenizer and suspended in 1 L methanol containing 0.1% hydrochloric acid. Ultrasonic extraction was performed three times for 30 min at room temperature. The extracts were combined, and the methanol was concentrated under reduced pressure to obtain extracts of *R. meyeri*. The extract was further purified with AB-8 macroporous resin to increase the anthocyanin content.

Naringenin (C15H12O5; MW: 272.25; purity: ≥98%; HPLC grade) was purchased from Chengdu Push Biotechnology Co. Ltd. (Sichuan, China) and stored at 2°C–8°C in a dry environment without light. The concentration of the stock solution was 100 mM in dimethyl sulfoxide (DMSO). The final DMSO concentration did not exceed 0.1% in the culture medium.

### Cell culture

mNSCs were harvested from the SVZs of 3-week-old and 23-month-old C57BL/6 mice. The mNSCs were maintained in dulbecco's modified eagle medium (DMEM) /F12 (Gibco, Gaithersburg, MD, USA) supplemented with basic fibroblast growth factor (bFGF) and human epidermal growth factor (hEGF).

### Cell viability assay

Cell viability was determined using a CCK-8 assay. Briefly, 5×10^4^ NSCs were seeded in 96-well culture plates and treated with various concentrations of *R. meyeri* anthocyanins and naringenin for 48 h and 72 h. CCK-8 solution was added to the 96-well plate (10 μL/well), followed by incubation at 37°C for 2 h. Cell viability was measured at 450 nm using a microplate reader.

### Cell cycle analysis

The Cell Cycle and Apoptosis Analysis Kit (Beyotime, Shanghai, China) was used to detect cell cycles according to the manufacturer’s instructions. Briefly, NSCs were collected by centrifugation, washed twice with cold PBS, and then fixed with 70% cold ethanol and stored at 4°C for 16 h. NSCs were then resuspended in a propidium iodide (PI) solution at room temperature in the dark for 30 min. All samples were washed and analyzed using a flow cytometer (BD 15 FACSVerse, BD Biosciences, San Jose, CA, USA). All conditions were performed in triplicate.

### Quantitative Real-time Polymerase Chain Reaction (qRT-PCR)

mRNA expression of a senescence-related gene (P16^ink4a^) was detected in NSCs using qRT-PCR. NSCs were seeded in six-well plates and cultured in medium containing various concentrations of Nar for 48 h. Total RNA was extracted using TRIZOL (Invitrogen, Carlsbad, CA, USA; Ct: 15596024) and was then reverse transcribed to cDNA using a reverse transcription kit (Takara Bio, Kusatsu, Japan; Ct: RR047A). SYBR green fluorescent dye (Bio-Rad, Hercules, CA, USA; Ct: 172-5124) was used for qRT-PCR. Relative gene expression levels were normalized to β-actin mRNA levels and calculated as 2^–ΔΔCT^. The primer sequences are listed in Table 1.

**Table 1:** Primer sequences used in this study.

**Genes**	**Forward Primers (5’-3’)**	**Reverse Primers (5’-3’)**
P16^INK4a^	GCGCTCTGGCTTTCGTG	CACTACCTTCTCCCGCCC
β-actin	GTGTTTCCTCGTCCCGTAG	AAAGTGGAGATTGTTGCCAT
Telomere	CGGTTTGTTTGGGTTTGGGTTTGGGTTTGGGTTTGGGTT	GGCTTGCCTTACCCTTACCCTTACCCTTACCCTTACCCT
36B4	CAGCAAGTGGGAAGGTGTAATCC	CCCATTCTATCATCAACGGGTACAA

### Measurement of telomere lengths

Telomere lengths were measured as previously described. Briefly, the amplification of the telomere repeats is called the T reaction, and that of the 36B4 gene is called the S reaction. The relative telomere ratio (T/S) = 2^–ΔΔCT^, ΔCT = CT(telomere) – CT(36B4). RT-PCR was performed to measure telomere lengths. The primer sequences are listed in Table 1.

### ROS assay

NSCs were treated with various concentrations of *R. meyeri* anthocyanins for 48 and 72 h, and then incubated with ROS reagent in the dark at room temperature for 30 min. ROS levels were determined using a multimode microplate reader. Intracellular and mitochondrial ROS were also observed under a fluorescence microscope.

### Neurosphere and differentiation assays

NSCs were derived from the mouse SVZ. Mice were sacrificed after anesthesia and the SVZ was carefully dissected and dissociated into single cells with 0.4 U/mL papain (Imgen, Alexandria, VA, USA; Cat No: LS003126). NSCs were plated in 10 cm dishes containing 10 mL NSC Basal Medium with 10 ng/mL EGF (PeproTech, Cranbury, NJ, USA; Cat No: AF-100-15) and 10 ng/mL FGF (PeproTech, Cranbury, NJ, USA; Cat No: AF-100-18B). Neurospheres were passaged every 5 days. NSCs from freshly dissociated passage-2 neurospheres were seeded on laminin (Gibco, Gaithersburg, MD, USA; Cat No: 23017015) and poly-L-ornithine hydrobromide (Poly-L; Sigma Aldrich, St Louis, MO, USA; Cat No: P3655)-coated glass slides in NSC Basal Medium without differentiation supplements. Differentiated cells were immunostained for TuJ1 (beta tubulin III; Thermo Fisher Scientific, Waltham, MA, USA; Cat No: 480011), MAP2 (Thermo Fisher Scientific, Cat No: MA5-12826), and glial fibrillary acidic protein (GFAP; Thermo Fisher Scientific, Cat No. PA5-16291), and then counterstained with 4′,6-diamidino-2-phenylindole (DAPI; Thermo Fisher Scientific, Cat No: D1306). Slides were examined using an Olympus BX53 microscope (Olympus, Madison, WI, USA).

Quantification of TuJ1- and MAP2-positive cells was performed by counting labeled cells from three independent experiments. Statistical analysis was performed using an unpaired *t*-test.

### Ki67 immunofluorescence staining

NSCs from freshly dissociated passage-2 neurospheres were seeded on laminin- (Gibco, Cat No: 23017015) and poly-L-coated glass slides in NSC complete medium with differentiation supplements. The experimental group was treated with Nar for 48 h. Next, the NSCs were immunostained with Ki67 (Abcam, Cambridge, UK; Cat No: ab15580) and counterstained with DAPI. Slides were examined using an Olympus BX53 microscope. Quantification of Ki67-positive cells was performed by counting labeled cells from three independent experiments. Statistical analysis was performed using an unpaired *t*-test.

### Morris water maze test

The learning and memory of female C57BL/6 mice (12 months old) were tested using the MWM, consisting of a round black pool that was 120 cm in diameter, 31 cm deep, and contained water at 23°C ± 1°C. The escape platform (11 cm in diameter, adjustable height) was placed in the center of one quadrant of the pool and hidden 1.5 cm below the water surface. Various prominent visual cues were set around the pool and remained in the same position during the training and testing periods. Each group was trained for 5 consecutive days facing three directions, and then tested on day 6 facing a new direction. In the test, the hidden platform was removed and free swimming was allowed for 60 s. The latency to escape from the water and the distance traveled to first find the platform were recorded using video-animal tracking software (EthoVision XT Base software, Noldus, Wageningen, Netherlands).

### Metabolomic analysis

Mass spectrometry data were collected using a Xevo G2-XS QTOF mass spectrometer (Waters, Borehamwood, UK) and processed using Progenesis QI (version 2.2; Waters) for liquid chromatography-mass spectrometry (LC-MS) data preprocessing. The metabolite identification was based on KEGG database sources (https://www.genome.jp/kegg/).

### RNA-seq analysis

RNA-seq was performed independently and uniformly for each sample. Clean reads were aligned to the reference gene sequence using bowtie-2, and the gene expression levels of each sample were calculated. DEG detection was conducted using the DEGseq method. The statistical results were based on the MA-plot method. The numbers of reads of specific genes obtained from the sample were sampled randomly, and the *P*-values were then calculated according to the normal distribution and corrected to *q*-values. To improve the accuracy of DEG detection, genes with a difference multiple of > 2 and a *q*-value of ≤ 0.001 were screened and defined as significantly DEGs. The RNA-seq data files that were generated in this study are available from the NCBI Gene Expression Omnibus (GEO) under the accession number GEO: GSE141342.

### ELISA

Blood samples were centrifuged at 1,000 × *g* for 30 min at 4°C, and the supernatant was collected and stored at –80°C until analysis. The protein levels of TNF-α were measured using a Mouse TNF-α ELISA Kit (Univ Company, Shanghai, China) according to the manufacturer’s instructions.

### Immunofluorescence images

Brain tissue was collected from mice after treatment with *R. meyeri* anthocyanins (*n* = 4 per group). Brain tissue was fixed with 4% paraformaldehyde in PBS overnight, followed by 20% and 30% graded sucrose dehydrations, each performed until the brain had sunk. Optimal cutting temperature compound was used to embed tissue samples, and successive coronal sections of 10 μm were cut using a freezing microtome. Tissue sections were washed with PBS and blocked for 1 h in 10% bovine serum albumin, 3% normal donkey serum, and 1% triton X-100 in PBS. Sections were then incubated with antibodies against nestin (Millipore, Burlington, MA, USA; Cat No: MAB5326), Ki67 (Abcam, Cat No: ab15580), TuJ1 (Covance, Princeton, NJ, USA; Cat No: MMS-435P-250), and GFAP (Abcam, Cat No: ab16997) overnight at 4°C. The next day, slides were washed three times and incubated with appropriate Alexa 488- and Alexa 568- secondary antibodies (Thermo Fisher Scientific) for 1 h at room temperature (1:1,000 dilution). DAPI staining was then used to label nuclei. Slides were examined using an Olympus BX53 microscope. Quantification of TuJ1-/nestin-positive cells was performed by counting labeled cells within the hippocampus from three independent experiments. Statistical analysis was performed using an unpaired *t*-test.

### Statistical analysis

Statistical analysis of the data was conducted using Graphpad Prism 5.0. Data are expressed as the mean ± standard deviation. Statistical comparisons of two groups were made using the unpaired *t*-test. Statistical comparisons of more than two groups were performed using analysis of variance. A two-tailed *P*-value of < 0.05 was considered statistically significant, and *P* < 0.01 was considered extremely statistically significant.

## Supplementary Material

Supplementary Figures

Supplementary Table 1
