# Supplementary material for: Anti-aging effects of Ribes meyeri anthocyanins on neural stem cells and aging mice
Source: Aging (Albany NY). 2020 Sep 12;12(17):17738–53. doi: 10.18632/aging.103955 (PMC7521483; doi:10.18632/aging.103955)
Supplement: Supplementary Figures [file aging-12-103955-s002..pdf]

SUPPLEMENTARY FIGURES

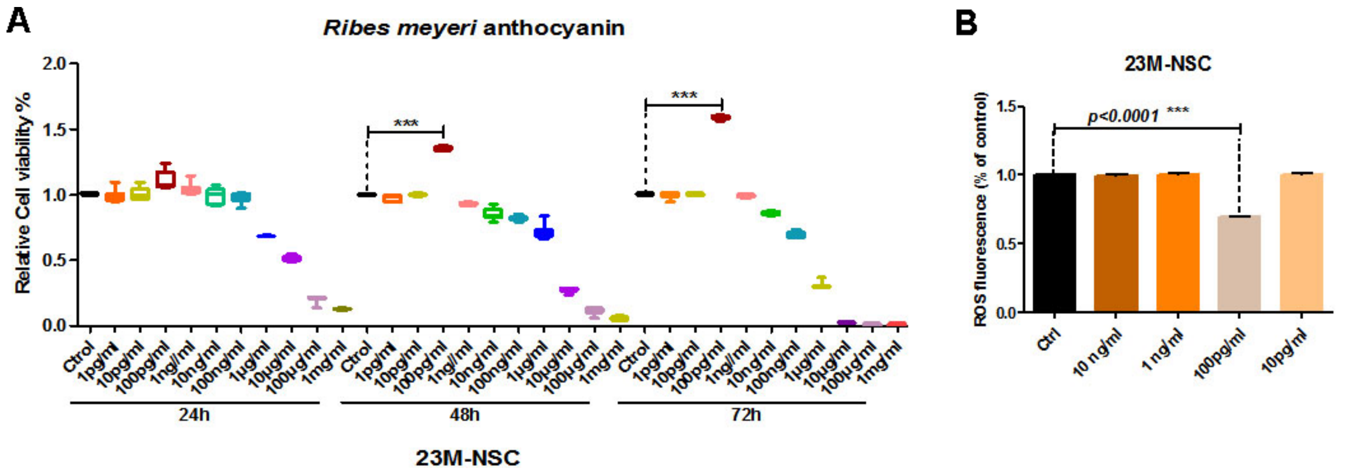

Supplementary Figure 1. *Ribes meyeri* anthocyanin concentration screening. (**A**) Optimal concentration of *R. meyeri* anthocyanins for promoting mouse neural stem cell (mNSC) proliferation. (**B**) Effects of 48 h of *R. meyeri* anthocyanin treatment on reactive oxygen species (ROS) levels. Data are presented as the mean ± SD of three independent experiments. *$P < 0.05$, **$P < 0.01$, and ***$P < 0.0001$ compared with untreated cells.

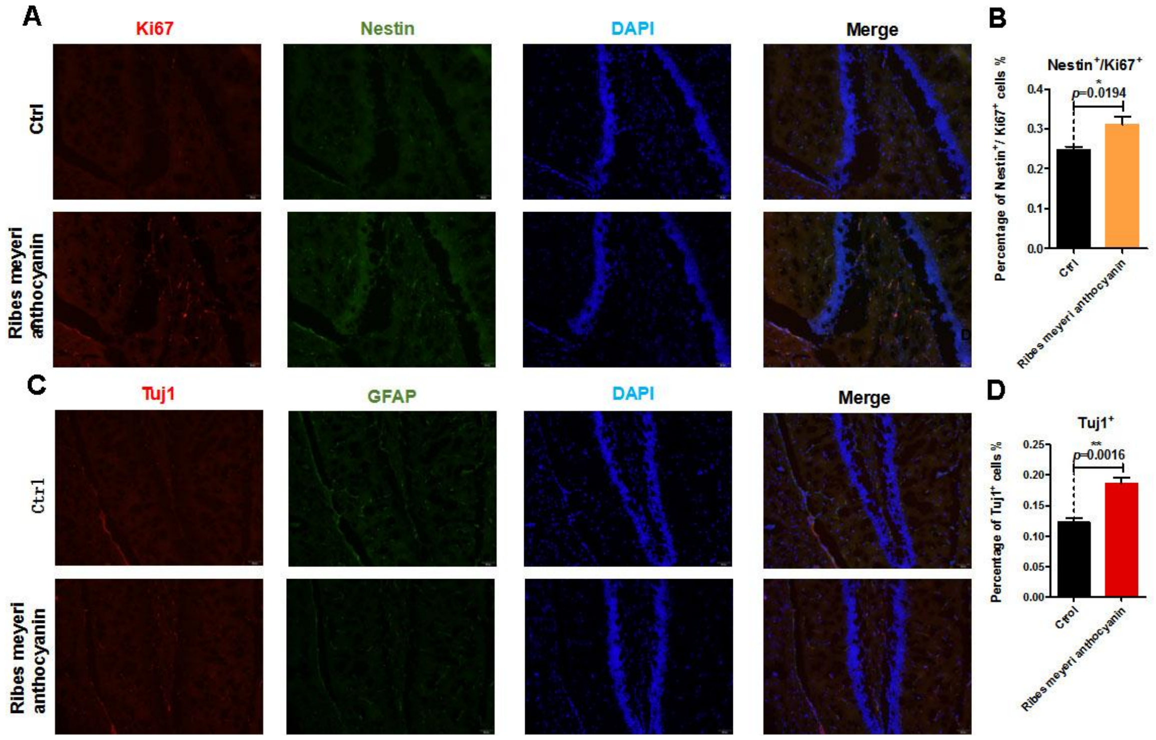

Supplementary Figure 2. *Ribes meyeri* anthocyanin treatment enhances the number of neural stem cells and neurons in mice *in vivo*. (**A**) Representative immunofluorescence images of nestin, Ki67, and nuclear (DAPI) staining in hippocampal regions of treated mice. (**B**) Percentage of nestin-/Ki67-positive cells in the hippocampus. (**C**) Representative immunofluorescence images of TuJ1, GFAP, and DAPI staining in hippocampal regions of treated mice (scale bar, 50 μm). (**D**) Percentage of TuJ1-positive cells in the treated groups, normalized to controls. Data are presented as the mean ± SD of three independent experiments. *$P < 0.05$, **$P < 0.01$, and ***$P < 0.0001$ compared with untreated mice.

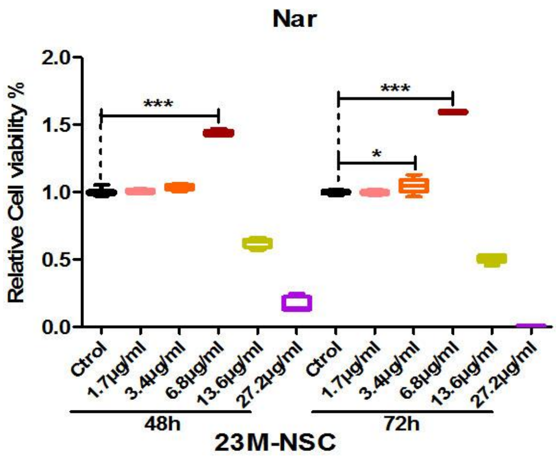

Supplementary Figure 3. Naringenin (Nar) promotes cell viability of mouse neural stem cells (mNSCs) as determined by CCK-8 assay. Data are presented as the mean ± SD of three independent experiments. *$P < 0.05$, **$P < 0.01$, and ***$P < 0.0001$ compared with untreated cells.
